# A Comparative Review of the Terms Epipharyngitis and Nasopharyngitis in Medical Literature

**DOI:** 10.7759/cureus.75738

**Published:** 2024-12-15

**Authors:** Abrahim N Razzak, Yuka Kaizu, Jay Starkey

**Affiliations:** 1 School of Medicine, Medical College of Wisconsin, Milwaukee, USA; 2 Department of Radiation Oncology, Tokyo Women’s Medical University, Tokyo, JPN; 3 Department of Radiology, Oregon Health and Science University, Portland, USA

**Keywords:** epipharyngeal abrasive therapy, epipharyngitis, epipharynx, long covid, medical english, nasopharyngitis

## Abstract

This review explores the usage of the term "epipharyngitis" in medical literature, particularly in non-English-speaking contexts. The term, although rarely used in contemporary English-language medical literature, may lead to confusion due to its overlap with more commonly used terms like "nasopharyngitis." This review aims to trace the origins of the term, analyze its usage across different languages, and discuss the implications of term differences in clinical practice and medical education.

## Introduction and background

"Epipharynx" as a term refers to a subsection of the pharynx described as the "post-choanal respiratory chamber continuous anteriorly with the nasal fossae and communicating inferiorly with the pharynx proper through the nasopharyngeal isthmus" [1]. The term corresponds to the upper pharynx, in contrast to the hypopharynx, which designates the lower portion of the pharynx below the pharyngoepiglottic fold at the level of the hyoid. Such a term is natural when considering terms in the context of other structures divided into upper, middle, and lower chambers, such as the epitympanum, mesotympanum, and hypotympanum. Indeed, "mesopharynx" is another term occasionally encountered [2]. Clinically, the term "epipharynx" is generally interchangeable with the term "nasopharynx," with nasopharynx being the most commonly used term for the location in Anglo-American medical literature. The term "epipharynx" can more commonly be found in anatomic or morphologic literature in which importance is placed upon specific locations and similarities/differences in comparative anatomy across different species. For example, a younger gorilla may have morphological differences in the epipharynx floors and hiatus nasopharyngeus in apposition with the laryngeal aditus [1].

Conflicts arise between anatomists and clinical scientists when the nasopharynx is emphasized by its nomenclature as an association with the nasal alimentary tract. However, the term "epipharynx" aims to highlight the tissue between the esophageal opening and the posterior location of the nasal cavity. Clinically, some have called this the post-nasal space to designate the portion of the respiratory tract specifically pointing to this location. However, much of the medical literature has continued to use the term "nasopharynx" canonically; yet, clinical pathologies within the nasopharynx are often associated with the nasal space, such as rhinorrhea from the common cold. In other words, the term "nasopharynx" is somewhat ambiguous because it can commonly refer to the more anterior nasal structures as well as those within the throat posteriorly. Cave, a British anatomist, was a proponent of changing the nomenclature due to its lack of specificity, especially when used in the context of non-Homo species morphology [1,3].

This subject becomes more nuanced when the epipharynx serves as the base term, with the addition of suffixes such as "-itis" (inflammation) or "-eal" (pertaining to). Given the widespread usage of "nasopharynx" as the de facto nomenclature of the region, the bulk of medical literature concerning the pathology of this anatomical region has concluded with "nasopharyngitis" or "nasopharyngeal" to refer to the region. However, there are select articles utilizing Hunter’s proposed anatomical landmark terminology in the nomenclature debates [1]. These refer to terms such as "epipharyngitis" or "epipharyngeal" to mark this region’s pathology. While not incorrect, it is not commonly used terminology and has often been employed within medical spaces of non-English-speaking audiences, notably in Japan.

Given the rarity and ambiguity of the newfound term "epipharyngitis" in modern English medical literature, the objective of this article is to review the existing literature on "epipharyngitis" and analyze the potential implications that can result from different terminologies, as well as propose a measure to unify treatment modules for "nasopharyngitis" and "epipharyngitis" in clinical literature.

## Review

Methods

The PubMed, Scopus, and Google Scholar databases were searched for pertinent publications dating back to 1865. Relevant search terms included "epipharyngitis," "epipharynx," "epipharyngeal," "nasopharyngitis," "nasopharynx," "nasopharyngeal," and "epipharyngeal abrasive therapy." Articles with redundant information and overlapping patient cohorts were excluded. Studies included were those that provided relevant information regarding the history of the terminology's use, recent treatment implications of the pathology, and associations between the terminologies. Ultimately, 23 publications were selected from the literature.

Results

Historical Context

As stated previously, "epipharynx" alone has been used more commonly in texts associated with zoological anatomy rather than medical annotations [1,3]. One may attribute this to the divide between human and animal biology; however, it is often agreed that the use of "nasopharynx" terminologically was canonized with the advent of the rhinoscope in the late 1800s, particularly when concerns arose about adenoid tumors of the nasopharynx [4,5]. After such descriptions, different pathologies of the "nasopharynx" were examined, including nasopharyngeal malignancies, myxomas, and sarcomas [6-8]. There is now a plethora of otolaryngology literature pertaining to the pathologies of the nasopharynx and nasopharyngitis. There was only one notable piece in 1948 by Dr. Theobold discussing the associated symptomatology of diseases within the vault of the epipharynx, which was also clarified as nasopharyngitis, and the importance of controlling recurring rhinitis [9]. It appears the de facto notation of "nasopharynx" was here to stay within the clinical literature.

That is, until 1960, when Horiguti, an otolaryngologist at Tokyo Medical and Dental University, published on the diagnostic and treatment utility of "epipharyngitis" instead of "nasopharyngitis." It is unclear what prompted the adoption of Hunter’s proposed nomenclature. However, the description of the pathology closely resembles what is recognized as nasopharyngitis or pharyngitis associated with the common cold. Horiguti then categorized the pathologies into acute, subacute, and chronic time courses, with significance placed on the presence of inflammatory cells, including eosinophils, in cases of "chronic epipharyngitis" [10]. Horiguti et al. were instrumental in the utilization of "epipharyngitis" in clinical literature, including various case reports associating the pathology with chronic allergic rhinitis, rheumatism, and headaches [11-13]. The crux of these reports was that treatment for latent/chronic epipharyngitis as a sole pathology led to improvements in comorbidities such as allergic rhinitis.

Current Usage

Currently, the term "chronic epipharyngitis" is more commonly used in the context of controlling inflammation of the structure. This inflammation can arise from various pathophysiological causes, including but not limited to posterior rhinorrhea, sore throat, chronic cough, local inflammation of the nasopharynx itself, autoimmune mechanisms such as vasculitis or nephropathies, or autonomic nervous system interactions such as sleep disorders, irritable bowel syndrome, or endocrine system diseases. It is thought to be a residual immune response that can cause both localized symptoms, such as postnasal drip, and systemic symptoms, such as chronic fatigue and headaches.

Horiguti, at the time, proposed a "B-spot therapy," a treatment consisting of a Lutze-type cotton swab soaked in a 0.5-1% zinc chloride solution applied to the epipharyngeal mucosa [11-14]. This later evolved into "epipharyngeal abrasive therapy" (EAT), wherein the treatment is conducted endoscopically to effectively address inflammation through both nasal and oral cavity approaches [14].

It is noteworthy, however, that there are currently no guidelines for chronic epipharyngitis or EAT, due to a lack of diagnostic standardization. Furthermore, the effectiveness of EAT varies based on the practitioner's skill and technique, and the procedure is not always easy to perform. While the Japanese Society of Oral and Pharyngological Sciences is working on evidence collection, it is believed that EAT stimulates the sympathetic and parasympathetic nervous systems through pharyngeal reflexes, endocrine system reflexes, and immune system reactions. This reactivation of autonomic functions is thought to produce therapeutic effects [14].

**Figure 1:**
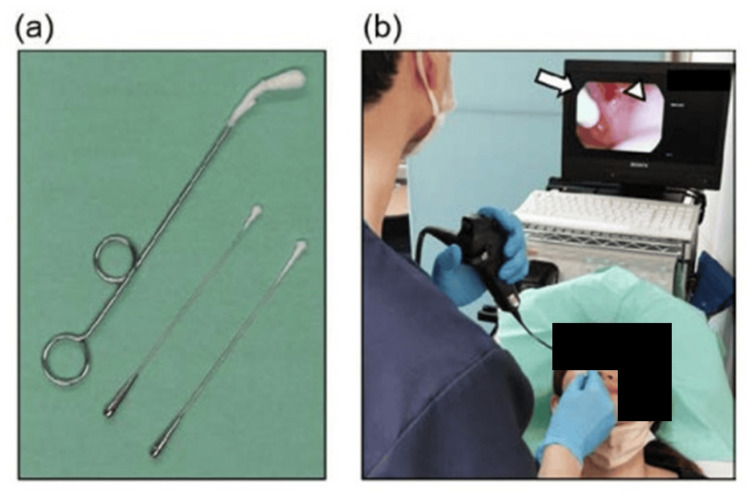
Visual representation of a typical EAT treatment (a) Cotton swab apparatus used for EAT; (b) swab soaked in 0.5% ZnCl₂ solution applied to the site indicated by the white arrow, with mild bleeding noted at the site marked by the white triangle. Figure consent obtained from Dr. Kazuaki Imai. EAT: epipharyngeal abrasive therapy

The treatment modality gained further attention when it was applied in the context of long-term coronavirus disease 2019 (COVID-19) symptoms. In one study, endoscopy was conducted on 58 patients who reported long COVID-19 symptoms such as fatigue, headaches, and attention disorders, with chronic epipharyngitis being a presenting pattern among those with long COVID-19 symptoms [15]. According to the report, there was a significant reduction in fatigue symptoms after EAT treatment, suggesting its positive effect in decreasing inflammatory cytokine secretions at the epipharynx (Figure 1) [15].

In another study, a comparison was made regarding the presence of angiotensin-converting enzyme 2 (ACE2) and transmembrane protease serine 2 (TMPRSS2) receptors, primary targets for severe acute respiratory syndrome coronavirus 2 (SARS-CoV-2), at the epipharyngeal mucosa [16]. The report found that EAT downregulates the expression of ACE2 and TMPRSS2, making it a potentially novel COVID-19 preventative method [16]. Another paper reported a downregulation of epipharyngeal abrasive CD4 T cells accompanied by symptomatic recovery [17].

Discussions

Based on the preceding discussion, while there is a clinical focus on chronic epipharyngitis as a distinct pathology treated with EAT, the terminology is not in common use. In contemporary medical literature, the term first appeared with Horiguti in the 1960s, and the continued utilization of "epipharyngitis" as a term has been largely confined to literature from Japan. Notably, no other countries have adopted this term, and it is possible that "chronic nasopharyngitis" has evolved as the de facto terminology for this pathology, albeit describing a wider anatomical space.

**Figure 2:**
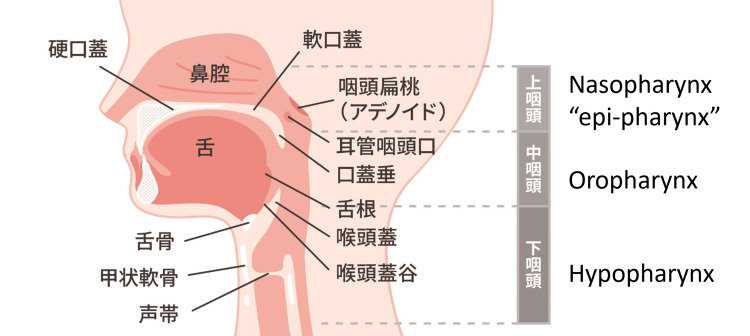
Visual representation of pharyngeal anatomy in Japanese, with main pharyngeal locations translated into English. Figure consent was obtained from Dr. Kazuaki Imai under the fair use clause of Creative Commons 2.0.

This may reflect a status quo bias, where the term persisted primarily within Japan, a non-English-speaking nation, or it may represent a deliberate decision by clinicians to focus on a specific anatomic or morphological subset, as suggested by Cave’s earlier remarks. Additionally, in Japanese, the term is written as “慢性上咽頭炎,” where the third character represents "up" or "superior" (Figure 2).

Linguistically, describing the term as "epi-" rather than "naso-" may provide a better understanding of its anatomical specificity. In Japan, the term "鼻咽頭" (nasopharynx), where the first character means "nose," is commonly used in contexts such as collecting samples for respiratory infectious diseases like COVID-19. However, the terms "epipharyngitis," "epipharynx," and "epipharyngeal" are also used in cancer-related contexts.

In the International Classification of Diseases, Tenth Revision (ICD-10) classification, pharyngeal neoplasms are divided into cancers of the nasopharynx, oropharynx, and hypopharynx [18]. In Japan, clinical studies on head and neck cancer use similar divisions but with different translations. "Nasopharynx" is translated as "上咽頭" (epipharynx), "oropharynx" as "中咽頭" (mesopharynx or middle pharynx), and "hypopharynx" as "下咽頭" (lower pharynx or laryngopharynx) [19]. These differences in terminology highlight potential variations in how anatomical demarcations are described in medical literature.

Although chronic epipharyngitis and nasopharyngitis are rare, other nations have proposed additional treatment options. For instance, in New Zealand, suction diathermy ablation has been recommended for nasopharyngeal crusting [20]. In Ukraine, immunorehabilitation therapy has been explored as a treatment for nasopharyngeal dysbiosis caused by Epstein-Barr virus infection, providing a conservative approach to improving symptoms without surgery [21]. From a radiological perspective, precise anatomical terminology is crucial for accurate communication between radiologists, clinicians, and surgeons when interpreting head and neck imaging studies. While both "epipharynx" and "nasopharynx" are valid terms in radiologic literature, their interchangeable use presents minimal clinical confusion as they refer to the same anatomical structure. This parallels other accepted anatomical synonyms in radiology, such as "dens" and "odontoid process." Notable radiology publications, including those in Radiographics from the Radiological Society of North America, occasionally employ the term "epipharynx," particularly in discussions of cross-sectional anatomy and fluoroscopic studies. This suggests that while "nasopharynx" remains the predominant term in radiologic practice, the use of "epipharynx" is recognized and understood within the field, especially when emphasizing specific anatomical relationships or in comparative imaging studies.

Given this context, Japanese otolaryngologists might consider adopting "chronic nasopharyngitis" as a standard term when aiming to disseminate treatment information to English-speaking audiences. While both terms are technically correct, "chronic nasopharyngitis" may align better with global terminology and reduce potential confusion. However, there is precedent for the use of the term “epipharynx.” One could argue that “epipharyngitis” could be easily adopted, and further that the use of "chronic nasopharyngitis" in Western literature also reflects a form of status quo bias.

Japan has a strong tradition of integrating modern Western medicine with traditional Japanese practices. In Japan, physicians are licensed to practice both modern Western medicine and traditional Japanese medicine (Kampo) under a single medical license. Kampo emphasizes maintaining the balance of bodily functions, such as immune and systemic balance. This approach may explain why treatments like EAT, which target latent infections or inflammation in the epipharynx, have gained wider acceptance in Japan.

Notably, this aligns with the focal infection theory, which suggests that localized infections, such as tonsillitis, can cause systemic diseases like arthritis. This theory has been recognized in Western medicine since the early 1800s [22]. For instance, immune system disorders caused by epipharyngitis are thought to be associated with IgA nephritis. One treatment for this condition, involving a tonsillectomy combined with steroids, was pioneered by Dr. Hotta in Japan [23].

## Conclusions

The debate between "epipharyngitis" and "nasopharyngitis" underscores the importance of anatomical precision and consistency in medical terminology. "Epipharynx" is primarily used in non-English-speaking countries, especially in Japan, while "nasopharynx" remains dominant in English medical literature. In Japan, unique treatments such as EAT and Kampo are employed for epipharyngitis; however, sufficient evidence or established guidelines are still lacking. Increasing awareness of these distinctions and standardizing terminology is crucial to improving diagnostic and treatment practices on a global scale.
